# Epidemiology of Antimicrobial Resistance in Complicated Acute Pyelonephritis—A Romanian Cohort Study

**DOI:** 10.3390/microorganisms14040767

**Published:** 2026-03-27

**Authors:** Marius-Costin Chițu, Daniel-Cosmin Caragea, Carmen-Marina Pălimariu, Teodor Salmen, Radu-Dragoș Marcu, Radu-Cristian Cimpeanu, Dan-Arsenie Spînu, Viorel Jinga, Anca Pantea Stoian, Dan Liviu Dorel Mischianu

**Affiliations:** 1 Doctoral School of “Carol Davila” University of Medicine and Pharmacy, 020021 Bucharest, Romania; marius-costin.chitu@drd.umfcd.ro; 2 Infectious Disease Department of Emergency University Central Military Hospital, 010825 Bucharest, Romania; carmen.palimariu@rez.umfcd.ro; 3 Cantacuzino National Military Medical Institute for Research and Development, 050096 Bucharest, Romania; dan.mischianu@umfcd.ro; 4 Department of Nephrology, University of Medicine and Pharmacy, 200349 Craiova, Romania; 5 3rd Department, Faculty of Medicine, Carol Davila University of Medicine and Pharmacy, 8 Eroii Sanitari Blvd., 050474 Bucharest, Romania; marcuradudragos@yahoo.com; 6 Urology Department, “Dr. Carol Davila” Central Military Emergency University Hospital, 134 Calea Plevnei, 010825 Bucharest, Romania; 7 Department of Emergency Medicine, Clinical Emergency County Hospital, 200642 Craiova, Romania; 8 Department of Urology, “Prof. Dr. Theodor Burghele” Clinical Hospital, 061344 Bucharest, Romania; viorel.jinga@umfcd.ro; 9 Department of Urology, University of Medicine and Pharmacy “Carol Davila” Bucharest, 020021 Bucharest, Romania; 10 Academy of Romanian Scientists, 050045 Bucharest, Romania; 11 Department of Diabetes, Nutrition and Metabolic Diseases, Carol Davila University of Medicine and Pharmacy, 050474 Bucharest, Romania; anca.stoian@umfcd.ro

**Keywords:** antimicrobial resistance pathogens, complicated acute pyelonephritis, *Escherichia coli*, local epidemiology

## Abstract

Antimicrobial resistance represents a major global challenge for healthcare systems, particularly in urinary tract infections (UTIs), where empirical antibiotic therapy is frequently required. Acute pyelonephritis (AP) remains a severe condition, requiring prompt diagnosis and treatment. Local epidemiological data are essential for optimizing therapeutic strategies. The aim of this study was to analyze the pathogen distribution and antimicrobial resistance (AMR) patterns in patients with complicated AP. An observational, analytical study on community-acquired and hospital-acquired AP was conducted on patients admitted with complicated AP between January 2021 and December 2025. After applying the inclusions and exclusions criteria, 553 urinary isolates with complicated AP were analyzed to determine pathogen distribution and phenotypic AMR patterns derived from antimicrobial susceptibility testing. A total of 109 (19.7%) AMR isolates presented resistance phenotype. Resistant phenotypes were more frequently observed among male gender; age did not reach statistical significance. This study highlights the continued predominance of *Escherichia coli* in complicated AP while demonstrating a significant AMR burden among non-*Escherichia coli* pathogens, particularly *Klebsiella* and *Pseudomonas* species. These findings emphasize the importance of local epidemiological surveillance and culture-guided antibiotic therapy in the management of complicated UTIs.

## 1. Introduction

Acute pyelonephritis (AP) is a severe infection of the upper urinary tract and represents a frequently encountered pathology in both inpatient and outpatient medical practice. The disease involves acute inflammation of the renal parenchyma and the pyelocaliceal system and may lead to serious complications such as sepsis, renal abscess, deterioration of renal function, or multiple organ failure. In many cases, AP requires hospitalization, parenteral antibiotic therapy, and close clinical monitoring [1,2].

Globally, *Escherichia coli* (*E. coli*) is the dominant etiological agent, accounting for 60–80% of community-acquired cases, followed by *Enterobacterales* such as *Klebsiella pneumoniae* (*K. pneumoniae*), *Proteus mirabilis* (*P. mirabilis*), *Enterobacter cloacae* (*E. cloacae*), and opportunistic pathogens such as *Pseudomonas aeruginosa* (*P. aeruginosa*) in complicated infections, respectively. Similar data have been reported in Romania, where *E. coli* accounts for approximately 70–75% of uropathogens; however, an alarming increase in ESBL-producing isolates, carbapenemase producers, and other complex resistance mechanisms has been observed [3,4,5]. Recent studies highlight a growing contribution of non-*E. coli* pathogens, which are disproportionately associated with antimicrobial resistance (AMR) and unfavorable outcomes [6].

AMR represents one of the most important global public health challenges. According to World Health Organization [7] and European Center for Disease Prevention and Control [8] reports, urinary tract infections (UTIs) caused by AMR organisms have increased significantly, reaching an estimated prevalence of 20–40% in certain regions worldwide. ESBL-producing and carbapenem-resistant *Enterobacterales* have become increasingly frequent, particularly in settings with high antibiotic consumption and suboptimal infection control practices [7,8,9]. AMR pathogens infections are associated with prolonged hospitalization, delayed clinical response, and the need for last-resort antibiotics, while excessive or prolonged antibiotic therapy further contributes to resistance selection [10].

The clinical impact of AMR is substantial, leading to prolonged hospitalization, increased rates of sepsis, the need for combination therapy, and the use of last-resort antibiotics (carbapenems, fourth-generation cephalosporins, aminoglycosides, colistin). Therefore, identifying epidemiological predictors of resistance is crucial for guiding empirical therapy and developing antimicrobial stewardship policies [8].

The early management of complicated AP relies largely on empirical antibiotic therapy, making accurate risk stratification essential. Recent evidence emphasizes the importance of integrating host factors and microbiological characteristics to predict treatment response and length of hospital stay, as illustrated by newly proposed composite clinical scores in UTIs [6]. At the same time, advances in molecular diagnostics and resistance phenotype profiling offer promising tools for early pathogen and resistance detection, although their availability remains limited in routine clinical practice [11].

In the international literature, male gender, advanced age, urological comorbidities, bladder catheterization, and recent antibiotic exposure are well-documented risk factors associated with AMR organism infections [12,13,14,15,16]. However, the prevalence of these risk factors varies significantly across geographic regions, making local data indispensable.

The aim of this cross-sectional retrospective study was to analyze the microbiological distribution of pathogens causing complicated AP and to evaluate phenotypic AMR patterns, as well as potential demographic factors associated with resistant isolates.

## 2. Materials and Methods

### 2.1. Study Design

A retrospective cross-sectional observational analysis was conducted in accordance with the Helsinki Declaration and received ethical approval by the Ethical Committee of the “Dr. Carol Davila” Central Military Emergency University Hospital, number 852/11 February 2026.

### 2.2. Study Setting

The study included consecutively adult inpatients admitted between 1 January 2021 and 31 December 2025 in the Inpatient Urology and Infectious Disease Department of Emergency University Central Military Hospital, with a clinical diagnostic of complicated AP.

### 2.3. Study Participants

Inclusion criteria consisted of adult patients with clinically and microbiologically confirmed complicated AP. Complicated cases included patients with obstructive uropathy, structural urinary tract abnormalities, or other clinical conditions requiring hospitalization.

Exclusion criteria included pediatric patients, patients with incomplete datasets, and cases with alternative diagnoses.

Data were collected retrospectively from hospital microbiological and laboratory database and included demographic variables (gender, age), microbiological data (identified pathogen), and AMR phenotypes.

**Figure 1:**
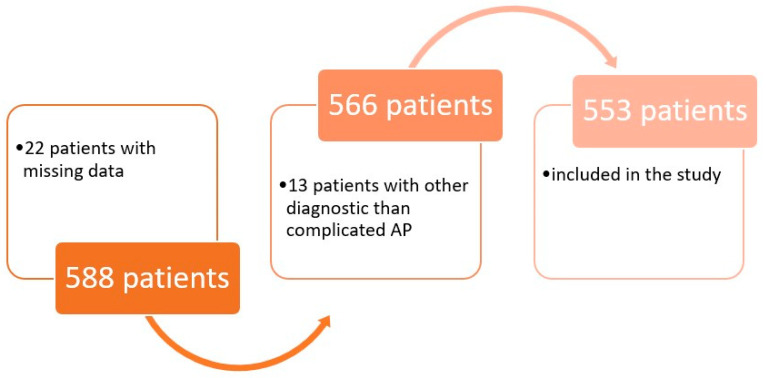
Flowchart of the included patient’s selection.

After evaluating the total of 588 included patients for the inclusion and exclusion criteria, we identified 22 patients with missing data and 13 patients with another diagnostic than complicated AP, so the data from the remaining 553 patients were evaluated, as shown in Figure 1.

The 553 patients with complicated AP that met the study inclusion criteria and were included in the final analysis, had one urinary isolate analyzed each. The cohort included both female and male patients across a broad adult age range. Demographic, biological, and microbiological data were linked at the individual patient level using the study database identifier.

The distribution of pathogens and resistance phenotypes was subsequently analyzed in relation to demographic and selected laboratory variables available in the retrospective database.

### 2.4. Microbiological Procedures

Urine samples were processed in the hospital microbiology laboratory using standard culture techniques. Bacterial identification was performed according to routine laboratory procedures.

Antimicrobial susceptibility testing (AST) was performed using standard phenotypic methods, including the disk diffusion method, and interpreted according to European Committee on Antimicrobial Susceptibility Testing [17] guidelines in use during the study period.

The resistance data analyzed in this study represent phenotypic resistance patterns derived from antimicrobial susceptibility testing, rather than molecular detection of resistance phenotypes.

AMR phenotypes (multi-drug-resistant (MDR), extensively-drug-resistant (XDR) and other) were classified according to the international definitions proposed by Magiorakos et al. [12].

### 2.5. Data Processing

The dataset was extracted and structured in Microsoft Excel databases-Variables included infectious agent, gender, age, and AMR phenotype. For statistic analyzing, the last variable was transformed into a binary variable indicating the presence or absence of resistant phenotype (1 = presence of resistance phenotypes and 0 = absence of resistance phenotypes)—including MDR, ESBL, or other clinically relevant resistance categories.

For the purpose of statistical analysis, different resistance categories (including ESBL production, MDR, and XDR phenotypes) were grouped into a single binary outcome to reflect the overall burden of clinically relevant AMR. While these categories differ in mechanisms and clinical implications, this approach was chosen to increase statistical power and allow a simplified exploratory analysis.

### 2.6. Statistical Analysis

Statistical analysis was performed using Microsoft Excel and GNU PSPP 1.4.1 software. To explore factors associated with AMR, a multivariable logistic regression model was constructed using a stepwise approach. The following variables were initially considered for inclusion based on clinical relevance and data availability: age, gender, selected biological parameters (including leukocyte count, neutrophil count, C-reactive protein, and fibrinogen), and microbiological variables (pathogen category).

Due to missing data and lack of statistically significant association in univariate analysis, most biological variables were not retained in the final multivariable model. The final model included age, gender and pathogen category (*E. coli* vs. non-*E. coli*), which were the most consistently available and clinically interpretable variables. Model fit was assessed using standard goodness-of-fit measures, and no major deviations were observed.

Descriptive statistics (including percentages, means, medians) and associations between categorical variables were analyzed using the chi-square test or Fisher’s exact test when appropriate. Continuous variables were analyzed using Student’s *t*-test or the Mann–Whitney U test depending on distribution.

Statistical significance was defined as *p* < 0.05.

### 2.7. External Validation

The obtained results, were compared with international datasets (World Health Organization [7], European Center for Disease Prevention and Control [8] and Centers for Disease, Control and Prevention [18]) and recent studies on AMR in AP.

Given the retrospective observational design and the limited availability of important clinical confounders, the identified associations should not be interpreted as causal relationships. The regression model reflects statistical associations within the available dataset and may be subject to residual confounding.

## 3. Results

### 3.1. Group Characteristics

The main baseline characteristics of the 553 patients are summarized in Table 1.

### 3.2. Distribution of Pathogens

**Figure 2:**
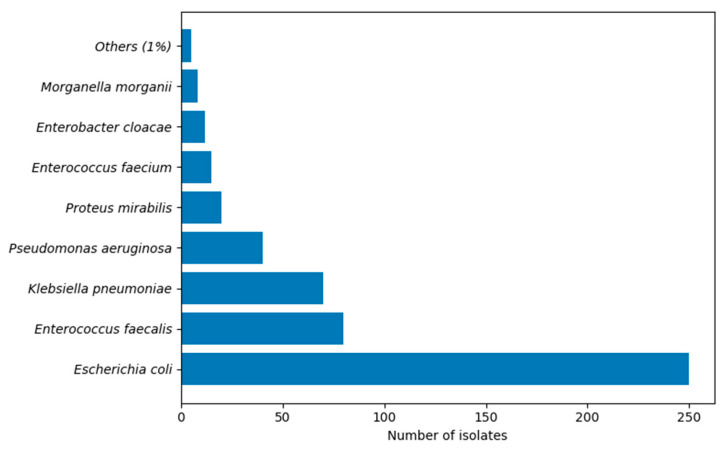
Distribution of Pathogens in Acute Pyelonephritis.

The distribution of the main species of the study cohort was as follows: *E. coli*—46.84%, *Enterococcus faecalis*—13.74%, *K. pneumoniae*—11.39%, *P. aeruginosa*—6.87%, as shown in Figure 2.

**Figure 3:**
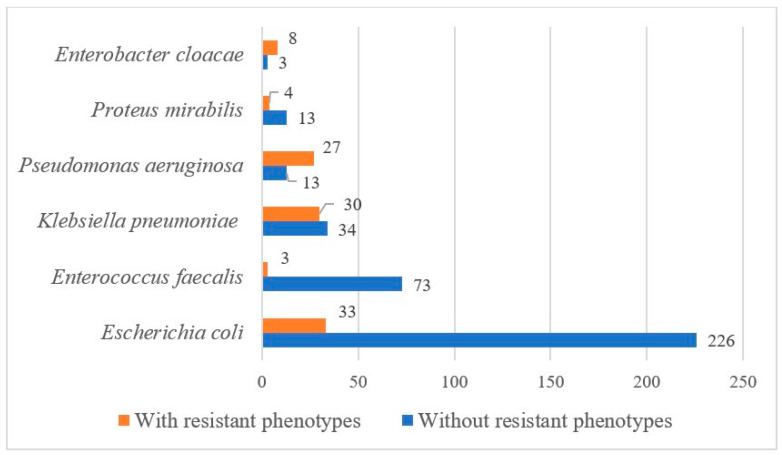
Combined Distribution of Pathogens and Resistance phenotypes in Complicated Acute Pyelonephritis.

From the total of 553 evaluated isolates, *E. coli* accounted for 46.8% respectively 259 cases, followed by *Enterococcus faecalis* with 13.7%, *K. pneumoniae* with 11.4%, and *P. aeruginosa* with 6.9%. Less frequently isolated pathogens included *P. mirabilis* with 3.1%, *Enterococcus faecium* with 2.3%, *E. cloacae* with 2.0%, *Morganella morganii* with 1.6%, and various opportunistic organisms with frequencies below 1%, as shown in Figure 3.

### 3.3. Prevalence of Resistance Patterns

In the analyzed cohort prevalence of resistant phenotypes was 19.7% of isolates harbored, which are distributed as follows: *P. aeruginosa*—65.8%, *K. pneumoniae*—46%, *E. cloacae*—27%, and *P. mirabilis*—23.5%, as shown in Figure 3.

In terms of resistance phenotypes, the highest proportion of isolates is *P. aeruginosa* with 67.5%, followed by *Klebsiella aerogenes* with 66.7%, *K. pneumoniae* with 46.88%, and, respectively, *P. mirabilis* with 23.5%.

*E. coli* demonstrated a comparatively lower resistance phenotype prevalence of 12.7%, while *Streptococcus group B*, *Staphylococcus aureus*, and, respectively, *Candida species* showed no detectable resistance phenotypes in this dataset.

**Figure 4:**
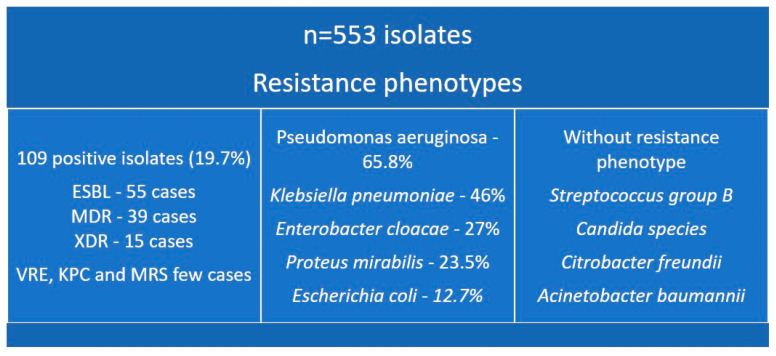
Conceptual overview of resistance distribution. ESBL—Extended Spectrum Beta-Lactamase; MDR—Multi-Drug-Resistant; XDR—Extensively-Drug-Resistant; VRE—Vancomycin-Resistant Enteroccoci; KPC—*Klebsiella pneumoniae* carbapenemase; MRS—Methicillin-Resistant *Staphylococcus aureus*.

Of the total isolates, 109 demonstrated identifiable resistance phenotypes. *P. aeruginosa* exhibited the highest proportion of phenotype-positive isolates (67.5%), followed by *Klebsiella aerogenes* (66.7%), *K. pneumoniae* (46.88%), and *Staphylococcus epidermidis* (100%, though based on a single isolate). *E. coli* showed a phenotype-positive percentage of 12.74%. The dominant resistance mechanisms included ESBL (55 cases), MDR (39 cases), XDR (15 cases), and isolated cases of VRE, KPC, and MRS. Notably, no resistance phenotypes were detected in several pathogens including *Acinetobacter baumannii*, *Candida species*, *Citrobacter freundii*, and *Streptococcus agalactiae*, as shown in Figure 4.

### 3.4. Distribution by Gender

**Figure 5:**
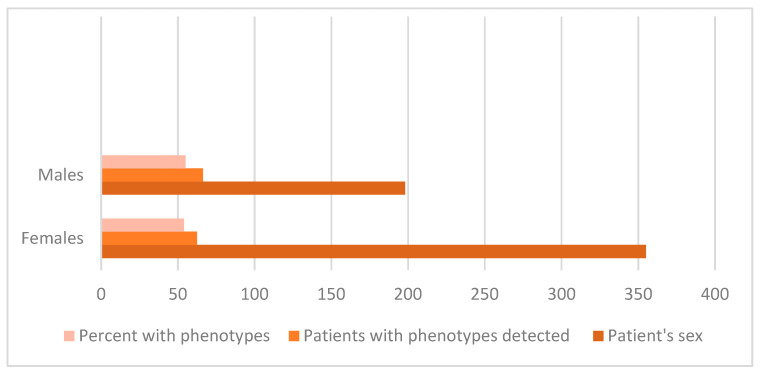
Quantitative representation included patients and detected pathogens.

Distribution by gender was established as it is represented in Table 2 and disposed in Figure 5.

Gender distribution data revealed that resistance phenotypes were present in 27.8% of isolates from males compared to 15.2% in females.

This difference was statistically significant (Fisher’s exact test *p* = 0.0005), indicating a higher proportion of resistance among male patients. Age-related analysis suggested that older patients had a slightly higher prevalence of resistance phenotypes, although this trend was not statistically significant.

Male patients remained statistically associated with AMR after adjustment for available variables (OR = 2.14), consistent with international literature attributing this risk to urological comorbidities and chronic colonization.

### 3.5. Role of Age

Mean age distribution of collected data and its relation with the detected phenotypes is shown in Table 3.

Regarding our curiosity about well-known and reported risk in elderly patients, we analysed collected data like “mean age with AMR phenotypes” (65.9 years), “mean age without AMR phenotypes” (63.3 years), resulting in a *p* value of 0.246, which represents a lack of statistically significant results. So, advanced age is a recognized risk factor in the literature; however, in this cohort, its effect was modest and statistically non-significant.

### 3.6. Multivariate Logistic Regression

The relationship between demographic variables and AMR was explored using logistic regression analysis.

Male patients showed a higher proportion of resistant isolates than female patients, (OR 2.04; 95% CI 1.1–2.69; *p* 0.017). Multivariable logistic regression analysis showed that male gender and infection with non-*E. coli* pathogens were associated with a higher resistant phenotype. However, given the limited availability of key clinical confounders, these findings should be interpreted with caution, as exploratory and hypothesis-generating rather than definitive causal estimates. Age was not significantly associated with resistant phenotype (*p* = 0.266), as shown in Table 4.

## 4. Discussion

Complicated AP remains a clinically significant infection associated with substantial morbidity, hospitalization, and frequent need for empirical antimicrobial therapy. In this context, the increasing burden of AMR represents a major challenge for clinicians managing severe UTIs.

Our results confirm and extend existing international data, providing novel insights into the AMR profile of complicated AP in a large cohort from Eastern European population.

### 4.1. Pathogen Distribution

In this cohort, *E. coli* remained the predominant pathogen, accounting for nearly half of all isolates, consistent with previous observational studies [3,4]. However, growing evidence indicates that infections caused by non-*E. coli* organisms, including *K. pneumoniae, P. aeruginosa* and *Enterococcus faecalis* are associated with more severe clinical outcomes, including increased rates of bacteremia, longer hospitalization, and higher treatment failure rates. For instance, comparative analyses have shown that *K. pneumoniae*-associated pyelonephritis may present with greater AMR and higher rates of systemic dissemination than infections caused by *E. coli* in community-acquired UTIs and AP [14,16].

This divergence aligns with international data indicating that empirical regimens targeted against *E. coli* may be insufficient in settings where non-*E. coli* pathogens account for a significant proportion of infections [19,20].

The inclusion of pathogen category (*E. coli* vs. non-*E. coli*) in the regression model was intended to explore differences in resistance burden between major pathogen groups. However, given the known relationship between pathogen type and resistance profiles, this variable may partly reflect intrinsic microbiological characteristics rather than independent patient-level risk factors.

Similar microbiological patterns have been reported in hospitalized cohorts of complicated UTIs, where structural urinary abnormalities, obstruction, or prior healthcare exposure may contribute to a broader spectrum of uropathogens. The presence of significant resistance patterns in *K. pneumoniae* and *P. aeruginosa* aligns with regional and international surveillance data indicating rising ESBL and MDR prevalence, particularly in Eastern Europe, as shown in Table 5 [21,22,23,24,25].

### 4.2. AMR in K. pneumoniae and P. aeruginosa

A considerable resistance burden was observed, particularly among *K. pneumoniae* with 46% and *P. aeruginosa* with 65.8% AMR, that could be due to the fact that these organisms are commonly associated with healthcare exposure and known for their ability to acquire resistance mechanisms. Consequently, their presence in complicated AP may have important therapeutic implications, particularly when empirical antimicrobial therapy is required prior to culture results.

These values are comparable to resistance rates reported in regions with a high AMR burden and reflect the increasing global dissemination of AMR Gram-negative organisms [11,12,22]. A numeric distribution of the resistant types of the included isolates is shown in Table 6.

Furthermore, the increasing prevalence of ESBL-producing *K. pneumoniae* in Eastern Europe has been linked to clonal expansion, horizontal phenotype transfer, and selective pressure from broad-spectrum antibiotic use, particularly during and after the COVID-19 pandemic. Nevertheless, the interpretation of these differences requires caution. The higher resistance rates observed in non-*E. coli* pathogens may not solely reflect organism-specific resistance patterns but could also be influenced by clinical context and patient-related factors [29]. Because several of these variables were not comprehensively available in the retrospective dataset used in this study, residual confounding cannot be excluded. These dynamics may partly explain the high resistance rates observed in this cohort.

The COVID-19 pandemic has likely accelerated these trends. Regional analyses from Eastern Europe and the Balkans have documented increased antibiotic consumption during the pandemic period, accompanied by a parallel rise in MDR urinary pathogens [20]. Disruptions in antimicrobial stewardship programs and widespread empirical antibiotic use have been proposed as major drivers of this phenomenon.

In the present cohort, male patients showed a higher proportion of resistant isolates compared with female patients. Logistic regression analysis suggested that male gender remained associated with resistant phenotypes after adjustment for the variables available in the dataset. However, this observation should be interpreted cautiously. The regression model could only incorporate variables available in the retrospective databases, and several clinically relevant confounders—including prior antibiotic exposure, healthcare contact, catheterization history, and urological interventions—were not available for analysis. Therefore, the observed association should be considered exploratory and hypothesis-generating rather than definitive evidence of an independent causal relationship [23].

Potential contributing factors known as being included in resistant antibiotherapy are: antibiotic exposure, repeated hospitalizations, biofilm formation, chronic colonization, and the presence of urological devices [30]. Biofilms allow bacteria to persist in hostile environments and facilitate the transfer of resistance phenotypes within microbial communities [22,31,32].

The lack of significant association between resistance and age mirrors findings in some studies but contrasts with others in which older age is a recognized risk factor for AMR organism infections.

The resistance phenotypes identified in this dataset—including ESBL, MDR, and XDR strains—therefore reflect the broader global challenge of AMR in UTIs. These findings highlight the limitations of traditional empirical therapies such as fluoroquinolones and third-generation cephalosporins in regions with a high prevalence of resistant organisms [19].

This underscores the importance of local epidemiology in guiding empirical antibiotic choices.

Comparisons with other Eastern European cohorts reveal similar patterns: high rates of ESBL-producing *Enterobacterales*, emerging carbapenemase producers, and a growing contribution from *P. aeruginosa* in complicated cases [12,21,22,23,24].

The low prevalence of resistance among streptococci and staphylococci is expected given their overall low representation in complicated AP.

### 4.3. Interpretation of Regression Models

Male gender was the only variable associated with AMR, whereas age did not demonstrate a statistically significant association. This observation suggests that host-related clinical factors may be more strongly associated with resistance patterns than demographic characteristics alone.

So, when we have a patient who is classically considered in a risk group, we should certify if it is like that or not. These results are evidence of how the approach based on medical practice is superior to the one based on paradigms.

Traditional epidemiological models have frequently identified advanced age as a risk factor for AMR pathogens infections. However, more recent analyses suggest that this association may be confounded by comorbidities, healthcare exposure, and prior antibiotic use rather than age itself [13,33].

The interpretation of the regression analysis requires particular caution. Although male gender and non-*E. coli* pathogens were associated with resistant phenotypes in the present model, these findings should not be interpreted as evidence of independent causal relationships. The retrospective design and the absence of several important confounders—including prior antibiotic exposure, hospitalization history, catheterization, and healthcare-associated infection—limit the ability to draw definitive conclusions. Therefore, the regression results should be considered exploratory and hypothesis-generating.

The present study provides valuable insights into the local epidemiology of pathogens and AMR patterns in complicated AP within a Romanian tertiary care setting. Such local epidemiological data remain essential for informing empirical treatment strategies, particularly in settings where resistance rates may vary substantially across regions and healthcare systems.

### 4.4. Clinical and Research Implications

The high prevalence of resistance among non-*E. coli* pathogens has important therapeutic implications. Empirical fluoroquinolone therapy may be inadequate in certain patient populations, particularly among male patients in this cohort. These findings align with current international guidelines that emphasize the importance of routine urine culture testing and susceptibility testing, adaptation of empirical therapy to local resistance patterns and consideration of a broader spectrum coverage in selected cases [19,24].

The observed resistance patterns must be interpreted in the broader context of post-pandemic antimicrobial consumption. Several regional studies from the Balkans and Eastern Europe have reported a marked increase in AMR urinary pathogens following the COVID-19 pandemic, attributed to empirical antibiotic overuse, disrupted stewardship programs, and increased hospitalization rates [29].

Emergency department-based epidemiological data from the United States further demonstrate a rising incidence of complicated UTIs and resistant organisms between 2016 and 2023, suggesting that this phenomenon is not geographically isolated [29]. These findings reinforce the notion that complicated AP is increasingly embedded within a global AMR crisis.

The resistance phenotypes identified in this study—particularly ESBL, MDR, and XDR—pose significant therapeutic challenges. Standard empirical options such as fluoroquinolones and third-generation cephalosporins may no longer provide adequate coverage in high-risk populations, necessitating the judicious use of carbapenems or combination therapy in selected cases [20,29]. However, such strategies must be balanced against the need to preserve last-line antibiotics and maintain antimicrobial stewardship principles [10].

Taken together, these findings emphasize the need for a paradigm shift in the management of complicated AP. The findings of this study support the importance of microbiological culture and susceptibility testing in the management of complicated UTIs. Whenever possible, antibiotic therapy should be guided by culture-based susceptibility results and adapted to local resistance epidemiology in order to optimize treatment outcomes and reduce unnecessary selection pressure for resistant organisms.

From a clinical perspective, male patients and those with suspected complicated AP should undergo prompt urine culture testing, early imaging when obstruction is suspected, and careful selection of empirical therapy tailored to local epidemiology [20,31].

### 4.5. AP in the Context of Complicated and Obstructive Disease

From the analyzed isolates, a considerable burden was observed, particularly among non-*E. coli* pathogens, male gender was the predictor of AMR (OR ≈ 2), age did not significantly determine AMR. The results emphasize the need for local guidelines and adjustment of empirical antibiotic therapy based on regional resistance profiles.

Beyond classical microbiological patterns, AP increasingly presents as part of a broader spectrum of complicated UTIs, particularly in patients with obstructive uropathy, structural abnormalities, or prior urological interventions.

Recent systematic analyses emphasize that complicated AP is associated not only with a broader etiological spectrum but also with more severe clinical presentations, delayed therapeutic response, and higher mortality rates [20,32].

Complicated AP represents a distinct clinical entity, in which urinary stasis promotes bacterial proliferation, ascending infection, and impaired antibiotic penetration. In this context, host-related factors and disease severity scores gain prognostic importance. Ishikawa et al. demonstrated that the qSOFA score exhibits good prognostic accuracy for in-hospital mortality in elderly patients with complicated AP, underscoring the importance of early risk stratification beyond microbiological data alone [29].

The present cohort, although lacking granular clinical severity data, showed a disproportionately high resistance burden among pathogens classically associated with complicated infections, such as *P. aeruginosa* and *K. pneumoniae*. This finding supports the hypothesis that a substantial proportion of cases likely belonged to the complicated AP spectrum, even in the absence of explicit obstruction or device-related metadata [20,32].

### 4.6. Study Particularities

In addition to anatomical factors, emerging research suggests that the urinary microbiome may influence susceptibility to recurrent or resistant infections. Recent microbiome studies have demonstrated that the urinary tract harbors diverse microbial communities even in healthy individuals, challenging the traditional concept of urinary sterility, while consistent with prior literature linking male gender to complicated UTIs, prostate-related urinary stasis, and higher rates of prior urological interventions [31,32,33]. Alterations in the urinary microbiome may facilitate colonization by AMR organisms and contribute to chronic infection or relapse.

Disruption of this microbial ecosystem—particularly through repeated antibiotic exposure—may facilitate colonization by AMR organisms. This phenomenon highlights the importance of antimicrobial stewardship and careful diagnostic evaluation.

Importantly, current clinical guidelines, including those from the Infectious Diseases Society of America, emphasize that asymptomatic bacteriuria should not be treated in most populations, as unnecessary antibiotic exposure may further disrupt microbial homeostasis and promote resistance [34]. Inappropriate treatment of colonization rather than true infection may partially explain the resistance patterns observed in certain high-risk subgroups.

The patterns observed in the present cohort are therefore consistent with a broader global epidemiological shift characterized by the progressive emergence of AMR Gram-negative pathogens in urinary infections. While *E. coli* remains the dominant etiological agent, surveillance programs consistently demonstrate that AMR pathogens is disproportionately concentrated among non-*E. coli* pathogens, particularly *K. pneumoniae* and *P. aeruginosa* [7,8]

This phenomenon has important clinical implications because empirical treatment strategies historically optimized for *E. coli* may fail to provide adequate coverage in regions where resistant *Enterobacterales* and non-fermenting Gram-negative bacilli contribute substantially to infection burden [14,19,35,36,37,38].

### 4.7. Future Directions

Future studies integrating clinical outcomes, comorbidities, and molecular characterization of resistance mechanisms would further enhance the understanding of AMR epidemiology in complicated AP.

The convergence of these mechanisms—horizontal phenotype transfer, clonal expansion, biofilm formation, and microbiome disruption—highlights the complexity of AMR organisms in urinary infections.

From a clinical perspective, these findings reinforce the importance of antimicrobial stewardship strategies aimed at optimizing antibiotic selection, reducing unnecessary antimicrobial exposure, and preserving the effectiveness of last-line agents such as carbapenems [18,39,40,41,42,43]. Additionally, such programs implementation is essential for optimizing antibiotic use through surveillance of antimicrobial consumption, adaptation of treatment guidelines, and strengthening microbiological monitoring, particularly in healthcare systems where resistance control infrastructure is still developing [42,43,44].

International guidelines increasingly emphasize the need for precision-guided therapy, integrating microbiological data, patient risk factors, and regional epidemiology in order to tailor empirical treatment strategies [45,46,47].

Future directions include integrating global epidemiological surveillance, expanding interdisciplinary stewardship strategies, and increasing patient involvement in the management of UTIs, considering the clinical burden of these infections and patients’ experiences with their treatment and outcomes [48,49,50].

In this context, surveillance studies such as the present investigation play a critical role in informing local treatment guidelines and improving clinical decision-making.

### 4.8. Study Limitation

Several limitations should be acknowledged. First, the retrospective design of the study limited the availability of certain clinical variables that may determine AMR, including prior antibiotic exposure, previous hospitalization, catheterization history, and healthcare-associated infection. Second, the microbiological analysis was based on phenotypic antimicrobial susceptibility testing, and molecular characterization of resistance mechanisms was not performed. Additionally, the use of a composite resistance outcome represents a limitation, as ESBL, MDR, and XDR phenotypes differ in both clinical significance and underlying mechanisms. Future studies should aim to analyze these categories separately in larger datasets. Finally, the study represents the experience of a single tertiary care center, which may limit the generalizability of the findings to other healthcare settings. Despite these limitations, the regression findings provide valuable insights into the epidemiology of AMR in complicated AP and may contribute to the development of improved predictive models for antimicrobial stewardship [51,52].

From a research standpoint, future studies should incorporate clinical severity scores, longitudinal outcomes, and molecular characterization of resistance mechanisms to refine predictive models and guide stewardship interventions.

## 5. Conclusions

Overall, the results reinforce the necessity of culture-guided therapy, ongoing local surveillance, and the implementation of antimicrobial stewardship principles. This microbiological analysis of the study cohort with complicated AP demonstrates a clear predominance of *E. coli*, alongside substantial resistance burdens among non-*E. coli* species. These findings highlight the importance of local microbiological surveillance and emphasize the role of culture-guided antimicrobial therapy in the management of complicated UTIs.

Adjustment of empirical antibiotic therapy based on regional resistance profiles is essential, particularly in high-risk groups, in order to ensure adequate treatment, limit the use of last-resort antibiotics, and support the rational administration of antimicrobial agents. Further studies incorporating broader clinical variables may help improve the understanding of resistance models in this patient population.

## Figures and Tables

**Table 1:** Baseline demographic, biological, and microbiological characteristics of the study cohort (n = 553).

Variable	n = 553
Age (mean ± SD)	63.8 ± 15.7 years
Age (median, IQR)	68 (55–75) years
Female (n, %)	355 (64.2%)
Leukocyte count (mean ± SD)	15,608 ± 5816 cells/mm^3^
Neutrophils (mean ± SD)	10,083 ± 4718 cells/mm^3^
Fibrinogen (mean ± SD)	747 ± 145 mg/dL
C-reactive protein (mean ± SD)	118.1 ± 108.5 mg/L
Most frequent pathogen—*Escherichia coli*	259 (46.8%)
*Enterococcus faecalis*	76 (13.7%)
*Klebsiella pneumoniae*	63 (11.4%)
*Pseudomonas aeruginosa*	38 (6.9%)
Other pathogens	117 (21.2%)
Isolates with resistant phenotypes	109 (19.7%)
ESBL phenotype	52 (9.4%)
MDR phenotype	37 (6.7%)
XDR phenotype	15 (2.7%)
Other resistance phenotypes (VRE, MRS, etc.)	5 (0.9%)

SD—Standard Deviation; IQR—interquartile range; ESBL—Extended Spectrum Beta-Lactamase; MDR—Multi-DrugResistant; XDR—Extensively-Drug-Resistant; VRE—Vancomycin-Resistant Enteroccoci; MRS—Methicillin-Resistant *Staphylococcus aureus.*

**Table 2:** Distribution by gender related to mean age and the percentage of detected phenotypes.

Gender	Total	With Phenotypes	Percent with Phenotypes	Mean Age (Years)
Females	355	54	15.21%	62.43
Males	198	55	27.78%	66.33

**Table 3:** Mean age distribution of collected data related to detected phenotypes.

Age Group	Total	With Phenotypes	Percent with Phenotypes
18–40	59	11	18.64%
41–65	164	31	18.9%
66+	330	67	20.3%

**Table 4:** Multivariate logistic regression for patient’s cohort.

Variable	Odds Ratio	95% Confidence Interval	*p*-Value
Age	1.01	0.99–1.02	0.266
Male gender	1.72	1.10–2.69	0.017
Non-*E. coli* pathogen	2.04	1.28–3.25	0.003

**Table 5:** Comparison of microbiological distribution and antimicrobial resistance patterns in complicated acute pyelonephritis across selected studies.

Study	Country	Population	Sample Size	Main Pathogen	Resistance Findings	Key Message
Present study	Romania	Complicated AP	553	*E. coli* (46.8%)	19.7% resistant phenotypes; ESBL and MDR present; High AMR in *K. pneumoniae* and *P. aeruginosa*	Non-*E. coli* pathogens show higher resistance burden
Flores-Mireles et al., 2015 [26]	USA	Complicated UTI	~1000+	*E. coli* predominant	Increasing AMR among Gram-negative pathogens	AMR is a growing challenge in complicated UTIs
Tandogdu et al., 2016 [27]	Europe	Complicated UTI cohorts	~800	*E. coli* dominant but diverse pathogens	High resistance among *Klebsiella* and *Pseudomonas*	Hospital-associated infections drive resistance
Foxman, 2014 [28]	USA	UTI epidemiology	Large cohorts	*E. coli* majority	Increasing AMR trends	Epidemiology varies by clinical context
Magiorakos et al., 2012 [12]	International	AMR definitions	NR	Multiple pathogens	Standardized MDR/XDR definitions	Framework for resistance classification
Medina, Castillo-Pino, 2019 [3]	Global	UTI epidemiology	NR	*E. coli*	Increasing Fluoroquinolones resistance	UTIs remain predominantly caused by *E. coli*, but AMR is increasing globally
Kang et al., 2018 [19]	Asia	Complicated UTI	NR	*E. coli*, *K. pneumoniae*	Rising ESBL prevalence > 20%	Empirical therapy increasingly challenged by ESBL-producing organisms
ECDC AMR Surveillance, 2023 [8]	Europe	Complicated UTI	Large cohorts	*Enterobacterales*	ESBL and carbapenem resistance	Regional heterogeneity with high AMR rates in Eastern Europe
WHO GLASS, 2023 [7]	Global	Complicated UTI	Large cohorts	*Enterobacterales*	MDR and carbapenemase spread	Global AMR expansion in urinary pathogens
Filev et al., 2025 [20]	Balkans	AMR pathogens in UTI	NR	*K. pneumoniae*, *P. aeruginosa*	AMR increase post-COVID	Pandemic-related antibiotic pressure increased resistance
Gao et al., 2026 [14]	China	UTI epidemiology	~3200	*E. coli*, *K. pneumoniae*	ESBL > 30%	Increasing resistance trends in retrospective surveillance

AP—acute pyelonephritis; UTI—urinary tract infections; *E. coli*—*Escherichia coli*; ESBL—extended spectrum beta-lactamase; MDR—multi-drug resistance; AMR—antimicrobial resistance; *K. pneumoniae*—*Klebsiella pneumoniae*; *P. aeruginosa*—*Pseudomonas aeruginosa*; NR—not reported; XDR—extended-drug resistance; WHO—World Health Organization; ECDC—European Center for Disease Prevention and Control.

**Table 6:** Numeric distribution of resistant isolates.

Phenotypes	Count
ESBL	55
MDR	39
XDR	15
VRE	1
KPC	1
MRS	1

ESBL—Extended Spectrum Beta-Lactamase; MDR—Multi-Drug-Resistant; XDR—Extensively-Drug-Resistant; VRE—Vancomycin-Resistant Enteroccoci; KPC—*Klebsiella pneumoniae* carbapenemase; MRS—Methicillin-Resistant *Staphylococcus aureus*.

## Data Availability

Data is available on request from the authors, due to the fact that this study is part of an ongoing doctoral project that has not finished publishing its results.

## Abbreviations

The following abbreviations are used in this manuscript:

AMR	Antimicrobial Resistance
AP	Acute Pyelonephritis
*E. cloacae*	*Enterobacter cloacae*
*E. coli*	*Escherichia coli*
*K. pneumoniae*	*Klebsiella pneumoniae*
MDR	Multi-Drug-Resistant
*P. aeruginosa*	*Pseudomonas aeruginosa*
*P. mirabilis*	*Proteus mirabilis*
UTIs	Urinary Tract Infections
